# Ground Vegetation in *Pinus sylvestris* Forests at Different Successional Stages following Clear Cuttings: A Case Study

**DOI:** 10.3390/plants11192651

**Published:** 2022-10-09

**Authors:** Dovilė Gustienė, Iveta Varnagirytė-Kabašinskienė, Vidas Stakėnas

**Affiliations:** Lithuanian Research Centre for Agriculture and Forestry, Liepų Str. 1, Kaunas District, LT-53101 Girionys, Lithuania

**Keywords:** Scots pine, clear-cut, percentage cover, species composition, forest floor, biomass

## Abstract

The impact of intensive forestry on various components of ecosystems has become the main subject of public and scientific debate in many regions in recent years. Forest ground vegetation is considered one of the most consistent and biodiversity-rich indicators of a certain stage of successional forest development. Therefore, changes in this forest component can potentially show the risks of forest damage due to clear-cutting and recovery trends. This study was carried out to identify the ground vegetation species diversity, including species composition and cover, also ground vegetation species relations with organic layer (forest floor) and upper mineral soil parameters at the different successional stages of the *Pinus sylvestris* L. stand development, including 1–2-year-old clear-cuts, and 6–130 years old stands. This study identified that the herb and dwarf shrub species were more light-demanding in the 2-year-old clear-cuts, as well as in the 6-year and 10-year old *P. sylvestris* stands compared to the middle-aged and mature forest stands. The dominant ground vegetation species, characteristic for the *Pinetum vaccinio-myrtillosum* forest type, were negatively dependent on the forest floor mass; they also had negative correlations with the concentrations of total P, K, Ca, and Mg in the forest floor and upper mineral soil but had positive correlations with the soil pH values and total N. The developed regression models of the percentage cover of mosses, herbs and dwarf shrubs according to the *P. sylvestris* stand age highlight the stabilization of the increase in the moss cover about 30 years after clear-cutting, with no clear trend for vascular species. The herbs and dwarf shrub species were highly variable during the stand rotation due to the species-specific characteristics and random factors rather than due to the influence of stand age. In this study, relatively short-term changes in ground vegetation species composition and percentage cover were determined after clear-cutting, but an important aspect is that new ground vegetation species appeared in the open areas, creating the potential for increasing species diversity. The clear-cutting system supports different species and numbers of herbs and mosses at different stages of stand development, which potentially increases the overall vegetation species diversity of the ecosystem.

## 1. Introduction 

Forest management of high intensity, such as clear-cutting, directly impacts vegetation species diversity at the ecosystem level [1]. Although clear-cutting has recently received a lot of interest and opposition from public organizations, this type of silvicultural system remains an important and effective practice in Lithuania [2,3]. In recent years, the areas of mature forest stands have varied in a range of 439–466 thou. ha, and about 19 thou. ha of mature stands was harvested annually by clear-cutting in Lithuania [3]. Moreover, clear-cutting accounted for 64% of the total area of felling in the State forests in 2021, and these fellings also prevailed in the *Pinus sylvestris* L. forests, especially in the private sector [3].

In the boreal and hemiboreal forest zone, the territories of managed mature forests are rapidly decreasing, and they are replaced by young forests after logging [4]. Overall, forest carbon balance and economic and social forest functions, as well as biodiversity, depend on disturbance regimes under different silvicultural systems and forest development stages [5]. 

Typically, clear-cutting leads to the complete removal of overstorey trees and partial removal of the understorey vegetation, and efforts are being made to retain the biodiverse trees and deadwood [6]. If final clear-cutting is applied, the mature forest site is drastically changed, forming new micro-climatic conditions for a new forest stand regeneration [7]. As a result, changes in forest structure, ecological properties, biodiversity, ecosystem resilience, and carbon storage are induced [8]. However, the Lithuanian Regulations for Forest Cutting (2010, updated in 2020) do not define how to preserve other components of forest structure, such as forest floor and ground vegetation, during clear-cutting [6].

Specifically, the understory vegetation is characterized by a higher species composition than the main tree layers and is likely more easily affected by microclimatic factors [9]. Early successional species, classified as colonists, stress-tolerant perennials, or pioneer species, are found in the forest only during the open-canopy stage, while late successional species, classified as competitive perennials, prevail after canopy closure [10]. Existing research recognizes the highest plant species richness at an early forest succession stage [11,12]. However, the forest-specific moss composition could be compared only with the species diversity found in the old managed forest [10,13].

Previous studies revealed that forest development is dependent on the soil nutrient and moisture regimes at an early stage of development [14]. Micro-environmental changes affect understorey vegetation differently: the light has a higher impact on the vascular plants, while throughfall precipitation on the nonvascular plants [15,16,17,18]. The abundance of understory vegetation also correlates with the increasing overstory cover, which reduces or suppresses understory plants [19]. Typically, the more intensive development of a phytocenosis is observed soon after clear-cutting, while the plant community change stabilizes in the long term [20]. Overall, the abundance of vegetation species increases at the beginning of the succession and tends to decrease in the later stages [21,22]. The highest understory vegetation cover is typically fixed in the 30-year-old stand [19]. However, this is still a matter of debate, and the response of understorey vegetation to clear-cutting could be site- and climate-dependent [23] or species-specific. Ref. [18] reported that the diversity of the understory communities is higher in the southern boreal forest with more abundant vascular species than in the northern boreal forests with a more important bryophyte layer. Nevertheless, there has been no detailed investigation of ground vegetation changes, typical for the hemiboreal forests, over the stand rotation following clear-cutting.

*P. sylvestris* forests were chosen for this study as typical forest sites recovering rather quickly in the clear-cutting sites at the hemiboreal forest zone [24,25]. Traditionally, *P. sylvestris* forests are managed as even-aged by the clear-cutting silvicultural system in Lithuania [3]. The understory vegetation associated with *P. sylvestris* varies considerably at an early successional stage following clear-cutting, and the initial effects of clear-cuttings have been recently reported [26,27]. 

This study aimed to determine the species diversity of the ground vegetation in the even-aged *P. sylvestris* stands at different forest development stages, including clear-cuts. More specifically, the objectives were to assess the species composition, percentage cover, and biomass of ground vegetation at the selected maturity ages of the stands, and to identify the relationships between ground vegetation species and the parameters of the organic layer (forest floor) and upper mineral soil layer. 

## 2. Results

### 2.1. Ground Vegetation Species Composition at Different Forest Succession Stages 

The clear-cuts of the former mature *P. sylvestris* stand were characterized by a high total species richness: 28 species in the 1-year-old and 47 species in the 2-year-old clear-cut were identified. A higher number of specific light-demanding species, such as *Senecio vulgaris* L., *Knautia arvensis* (L.) Coult, *Conyza canadensis* (L.) Cronquist, and *Polygonum maculosa* L. were found in the clear-cuts compared to the mature *P. sylvestris* stand (Table 1). In the 6-year stand, the number of atypical species diminished, and the total species richness decreased to 25 species. The peak of *P. sylvestris* forest-related but light-demanding species continued until a 10-years old forest stand, and 31 species were observed at this age of the stand. The decreasing trend of species richness starts in the 15-year stand, and the number of species was in the following order: 28 species (15-year stand), 25 species (30-year stand), 17 species (70-year stand), and 12 species (mature forest) (Table 1). 

**Figure 1:**
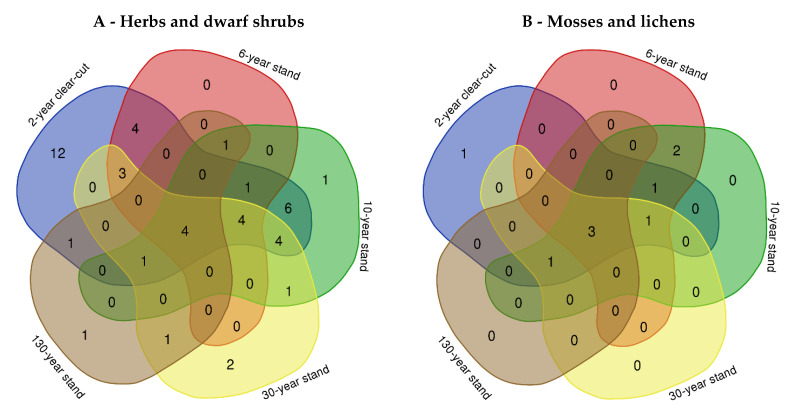
Venn diagram showing the diversity and overlap of the herbs and dwarf shrubs (**A**), mosses, and lichen (**B**) at the selected *Pinus sylvestris* sites, taken to represent clear-cuts (2-year-old clear-cut), young stands (6-year and 10-year stand), middle-aged stands (30-year stand) and mature stands (130-year stand).

The Venn diagram revealed that 12 species of herbs and dwarf shrubs were indicated as unique species for the 2-year-old clear-cut (Figure 1A). These species were *Succisa pratensis* Moench, *Salix caprea* L., *Hypochaeris radicata* L., *Leontodon hispidus* L., *Convolvulus arvensis* L., *Potentilla arenaria* Borkh., *Plantago lanceolata* L., *Conyza canadensis* (L.) Cronquist, *Anthoxanthum odoratum* L., *Trifolium repens* L., *Polygonum maculosa* L., and *Convallaria majalis* L. (Table 1). Six species overlapped in the 2-year-old clear-cut and 10-year forest stand, and those species were *Verbascum thapsus* L., *Hypericum perforatum* L., *Viola canina* L., *Rubus caesius* L., *Epilobium parviflorum* Schreb., and *Trientalis europaea* L. (Figure 1A, Table 1). Four species were detected in 2-year-old clear-cut and 6-year stand, and those species were *Senecio vulgaris* L., *Pteridium aquilinum* (L.) Kuhn, *Knautia arvensis* (L.) Coult, and *Chamaenerion angustifolium* (L.) Holub. Four species, *Vaccinium vitis-idaea* L., *Juniperus communis* L., *Luzula pilosa* (L.) Willd, and *Vaccinium myrtillus* L., were found in the 2-year-old clear-cut, 6-, 10-, 30-, and 130-year stands. Additionally, four species, *Festuca ovino* L., *Betula pendula* Roth., *Rubus idaeus* L., and *Calluna vulgaris* (L.) Hull, overlapped in the 2-year-old clear-cut, 6-, 10-, and 30-year *P. sylvestris* stands. When comparing 2-year-old clear-cut, 6-, 10-, 30-, and 130-year stands, only the species *Chimaphila umbellata* (L.) W. P. C. Barton was identified as a unique species in the mature stands (Figure 1A, Table 1).

One moss species (*Polytrichum juniperinum* Hedw.) was found in the 2-year-old clear-cut. A total of three species of mosses (*Hylocium splendens* (Hedw.) Schimp., *Dicranum polysetum* Sw.*,* and *Pleurozium schreberi* (Brid.) Mitt.) were obtained in the 2-year-old clear-cut, 6-, 10-, 30-, and 130-year stands (Figure 1B, Table 1). In the 6- and 10-year stands, one lichen, and one moss species overlapped, and these species were *Cladonia fimbriata* (L.) Fr. and *Rhacomitrium canescens* (Hedw.) Brid. No exceptionally specific moss species were obtained in the mature forest stand. 

### 2.2. Ground Vegetation Cover at Different Forest Succession Stages

The assessment of percentage coverage of all ground vegetation species showed that the mosses were the dominant vegetation, covering more than 89% in the 30- and 70-year stands, and mature forests (Table 2). The most frequent moss species in the mature stand were *Hylocium splendens (Hedw.) Schimp*. (53.7%), *Pleurozium schreberi* (Brid.) Mitt. (20.9%), and *Ptilium crista-castrensis* (Hedw.) De Not. (14.1%) (Table 1). In the 30- and 70-year *P. sylvestris* stands, *Pleurozium schreberi* prevailed with the coverage of 50.7% and 67.8%, respectively, followed by *Hylocium splendens* (30.3% and 13.7%), and *Ptilium crista-castrensis* (5.6% and 10.2%). The clear-cutting tended to decrease the moss cover at the early successional stage from 56% in the 1-year-old clear-cut to 18% in the 2-year-old clear-cut, and 25% in the 6-year stand (Table 2). In the 2-year-old clear-cut, new species such as *Pohlia nutans* (Hedw.) Lindb., *Polytrichum commune* L., and *Polytrichum juniperinum* Hedw. were detected. Furthermore, *Pohlia nutans* with a cover of 13.9% was fixed as dominated in the 6-year stand (Table 1). The coverage of the mosses recovered up to 53.0% and 59.8% at the 10-year and 15-year stand, respectively (Table 2). *Pleurozium schreberi* (33.6%) prevailed in the 10-year stand, and *Cirriphyllum piliferum* (Hed.) Grout. (29.0%) in the 15-year stand (Table 1).

Among the species of herbs and dwarf shrubs, *Vaccinium* *myrtillus* and *Vaccinium vitis-idaea* were the dominants with the highest percentage cover of 10.6% and 5.8%, respectively, in the mature *P. sylvestris* stand, representing *Pinetum vaccinio-myrtillosum* forest type (Table 1). In comparison to the mature forest stand, the lower total coverage (14,5%) of dwarf shrubs was found in the 1-year-old clear-cut, and the next year, in the 2-year-old clear-cut, the coverage of herbal plants increased (23.9%) (Table 2). *Vaccinium myrtillus* and *Vaccinium vitis-idaea* remained the most common species with the highest prominence value in the 1-year-old clear-cut (Table 1). The coverage of *Vaccinium myrtillus* and *Vaccinium vitis-idaea* was 6.6–6.8% and 1.9–2.2%, respectively, in the 1–2-year-old clear-cuts. The coverage of *Pteridium aquilinum* was 3.5–10.7 times higher in the 1–2-year-old clear-cuts than in the mature *P. sylvestris* forest. Some specific species, such as *Luzula pilosa*, *Senecio vulgaris*, and *Trientalis europaea*, were observed in the clear-cuts. The highest prominence value for *Vaccinium myrtillus* and *Senecio vulgaris* was fixed in the 2-year-old clear-cut. Significant differences in the species composition were observed in the 6-year stand, where *Calluna vulgaris* was the most frequent and dominant species with a coverage of 25.9% (Table 1). Other common species obtained were *Vaccinium vitis-idaea*, *Rubus idaeus*, *Pteridium aquilinum*, *Festuca ovino*, and *Vaccinium myrtillus*. The highest coverage of herbs and dwarf shrubs (59.0%) was found in the 10-year stand (Table 2). In this stand, the most frequent species were *Calluna vulgaris* (23.4%), *Vaccinium vitis-idaea (*13.4%), and *Vaccinium myrtillus (7*.8%) (Table 1). The species *Calluna vulgaris* had the highest prominence value in the 6-year and 10-year stands. In the 10-year stand, the coverage of both *Vaccinium* *myrtillus* and *Vaccinium vitis-idaea* was higher in the 6-year stand. *Vaccinium vitis-idaea* was identified as the most frequent in the 15-year and 30-year stands, covering 24.8% and 6.3%, respectively, and it was found in all research sites. *Calluna vulgaris*, covering 2.3%, was identified as the less frequent species, found in 25% of the plots of the 15-year stand (Table 1). In the 15-year stand, the frequent species were *Festuca ovino*, *Melampyrum pratense* L., and *Vaccinium myrtillus.* In the 30-year stand, the coverage of *Vaccinium vitis-idaea* was followed by *Vaccinium myrtillus, Festuca psammophila* (Hack. ex Čelak.) Fritsch*,* and *Luzula Pilosa.* In the 70-year stand, both *Vaccinium* *myrtillus* and *Vaccinium vitis-idaea* were found in all research plots, but *Vaccinium myrtillus* coverage was higher than *Vaccinium vitis-idaea*.

**Figure 2:**
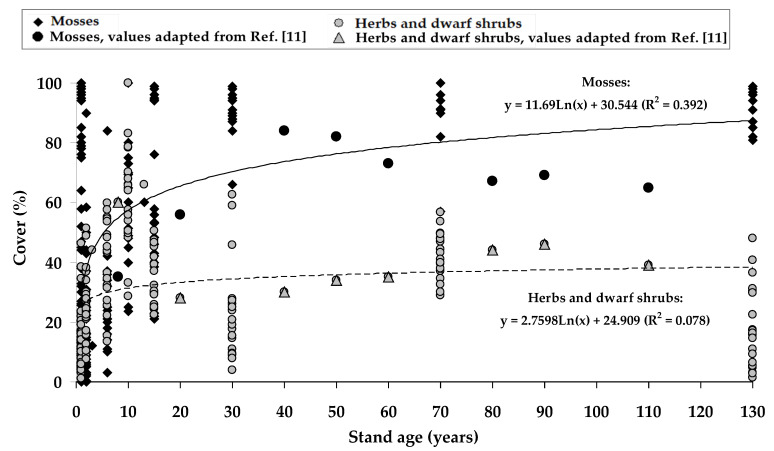
Chronosequence models for *Pinus sylvestris* stand development based on regression modeling of mosses and herbs and dwarf shrubs. Some intermediate data-points were adapted from [11].

The regression models of the percentage cover of mosses, in addition to herbs and dwarf shrubs, according to the *P. sylvestris* stand age, were developed (Figure 2). Regardless of the successional status of individual species, the increase in the moss cover stabilized approximately 30 years after clear-cutting. Despite the relatively high variability of the data, the faster growth of herbs and dwarf shrubs was fixed at the early stage of the stand; this trend changed insignificantly in the 30–50-year-old stand and showed a slight decrease trend in the mature forest.

The plant community obtained in 2-year-old clear-cut has a higher Shannon species diversity index value and was assigned as the most diverse in comparison with the 1-year old clear-cut and the *P. sylvestris* stand of 6–130 years old (Table 3). The lowest diversity of the plants was identified in the 70-year stand. The obtained results were comparable with the total species richness values obtained at all studied sites (see Table 1). However, the highest equitability was found for the 10-year stand followed by the 2-year-old clear-cut and 6-year stand. 

For herbs and dwarf shrubs, the highest Ellenberg indicator values for light were obtained in the 2-year-old clear-cut, also in the 6-year and 10-year *P. sylvestris* stands, followed by a consistent downward trend towards the oldest studied stands (Table 4). The Ellenberg indicator values for temperature inconsistently increased, while the values for the soil moisture showed a decreasing trend with increasing stand age. The Ellenberg values for nitrogen were higher in the 1–2-year-old clear-cuts compared to the older stands.

For mosses, there were no clear trends of Ellenberg indicator values for light, temperature, moisture, and pH over the entire stand rotation (Table 4). However, the highest Ellenberg indicator values for light and soil moisture were fixed in the 10- and 15-year stands. The values for pH were higher in the stands older than 10 years old compared to the stands of an early stand development stage. 

### 2.3. The Ground Vegetation Relations with the Forest Floor and Mineral Soil Parameters

**Figure 3:**
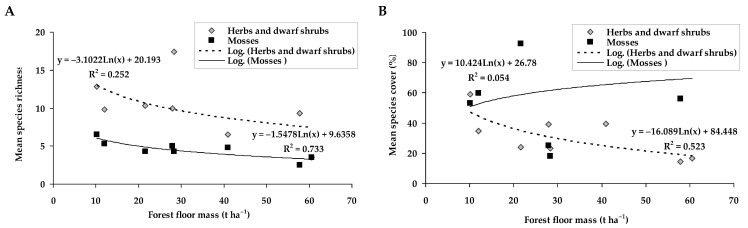
Relations between species richness (**A**) and cover (**B**) of herbs and dwarf shrubs and mosses with the forest floor mass; *p* ≤ 0.05.

The species richness of the mosses had relatively strong relation with the forest floor mass (*R^2^* = 0.733), i.e., the lower number of the mosses was identified under the higher forest floor mass (Figure 3). The species richness of herbs and dwarf shrubs also showed a downward trend with the increase in forest floor mass. The higher forest floor mass tended to decrease the cover of herbs and dwarf shrubs, while no relation with the moss cover was obtained.

The cover of *Vaccinium myrtillus* moderately (0.50 ≤ *r* ≤ 0.75) positively correlated with forest floor pH and total N (Table 5). The moderately negative correlations were found between the cover of *Vaccinium myrtillus* and organic C, total P, K, Ca, and Mg in forest floor, and total P, K, Ca, and Mg, in addition to P_2_O_5_, K_2_O, and mobile Ca in the upper (0–10 cm) mineral soil layer. The cover of *Vaccinium vitis-idaea* moderately positively correlated with the pH value obtained in the forest floor and upper mineral soil layer, as well as with the organic C and total N of the mineral soil (Table 5). Moderately negative correlations were found between the *Vaccinium vitis-idaea* and total P, K, Ca, and Mg obtained in both forest floor and mineral soil, as well as with P_2_O_5_, K_2_O, and mobile Ca of mineral soil.

Other ground vegetation species were less related to the chemical parameters of the forest floor and upper mineral soil (Table 5). However, for example, *Luzula pilosa* showed a moderately (0.50 ≤ *r* ≤ 0.75) positive relationship with forest floor pH and a weak (0.30 ≤ *r* ≤ 0.50) relationship with organic C and total N of the forest floor. Moderately negative correlations were found between the cover of *Luzula pilosa* and total P, K, and Ca obtained in both forest floor and upper mineral soil layer, as well as with total Mg, P_2_O_5_, and mobile Ca obtained in mineral soil. The relationship between the cover of *Hylocium splendens* with total P, K, and Ca in forest floor and mineral soil, and also with total Mg, K_2_O, and mobile Ca and Mg in mineral soil mostly showed a correlation coefficient about *r* = 0.5. Similar relationships were identified between the cover of the *Ptilium crista-castrensis* and *Dicranum polysetum* with the chemical parameters of the forest floor and upper mineral soil layer.

### 2.4. The Ground Vegetation Biomass

The highest total biomass of ground vegetation was found in the 10- and 15-year *P. sylvestris* stand (Table 6). In the 30-, 70- and 130-year stands, the total biomass of ground vegetation was 1.6–3.6 times lower than in the 10- and 15-year stands. The total biomass of total ground vegetation in the 6-year stand was different from the remaining values. The highest mass of mosses was obtained in the 1-year-old clear-cut, which was comparable with the values obtained in the mature *P. sylvestris* stand. The lowest mass of mosses was found in the 2-year-old clear-cut, and then increased consistently up to the 30 years age. The biomass of herbs and dwarf shrubs showed large variability, but the highest values were obtained in the 10- and 15-year stand. The obtained results indicated that the biomass of herbs and dwarf shrubs tended to be more species-specific than depended on stand age. 

**Figure 4:**
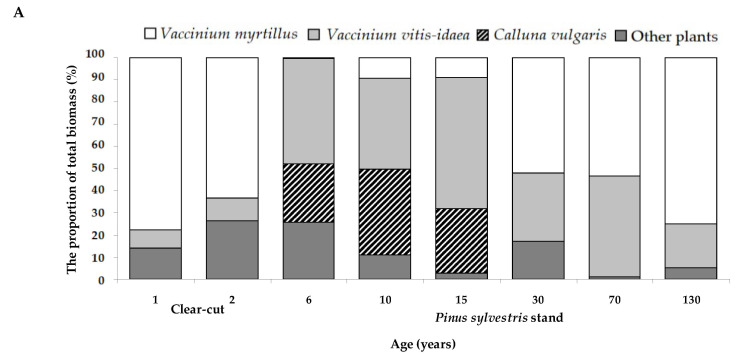

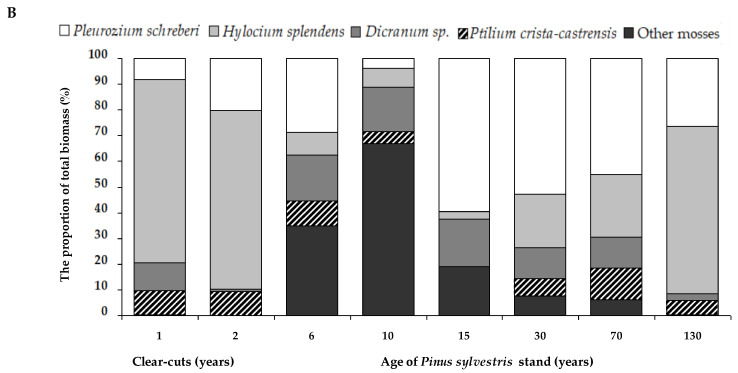
The proportion (%) of dominant herbs and dwarf shrubs (**A**) and moss (**B**) species of the total biomass of dominated ground vegetation species at different *Pinus sylvestris* sites, including 1–2-year-old clear-cuts and 6–130 years old stands.

The herbs and dwarf shrubs in different sites consisted of similar species groups with the highest biomass proportion of the dominant species *Vaccinium myrtillus* and *Vaccinium vitis-idaea* with slight exceptions at the 6-year stand (Figure 4A). In the period from 6- to 15-year stand, a larger proportion of *Calluna vulgaris* biomass was observed. Mosses consisted mainly of the same species groups with the highest biomass proportion of the *Pleurozium schreberi, Hylocium splendens,* and *Dicranum* sp. (Figure 4B).

## 3. Discussion

Ground vegetation species richness and diversity varied between different study sites, including 1–2-year-old clear-cuts, and the *P. sylvestris* stands of different ages recovered after clear-cutting. The results of this study indicated that the most abundant moss species were *Hylocium splendens*, *Pleurozium* *schreberi*, and *Ptilium crista-castrensis* (see Table 1), and well represented the moss species typical for the *Pinetum vaccinio-myrtilliosa* forest type [11,28]. Previous studies also stated that *Ptilium crista-castrensis* and *Hylocomium splendens* were dominated species in the comparable *P. sylvestris* forest stands [29]. The dominant species of vascular plants were *Pteridium aquilinum* at an early stage after clear-cutting and both, *Vaccinium* *myrtillus* and *Vaccinium vitis-idaea*, which were obtained at all studied sites of different stand ages (see Table 1). Total species richness tended to increase at the clear-cuts, and this lasted up to 10 years after clear-cutting. However, the species of vascular plants showed a decreasing trend, and gradually the prevailing moss species was recorded. The predominance of light-tolerant vascular plants in the boreal forest zone was identified by several studies [30,31]. 

Our findings were consistent with previous studies that showed differences in ground vegetation cover and species diversity along a stand age gradient [10,11,18]. The species composition and coverage vary since the vegetation colonizes the available resources after clear-cutting-induced disturbance and consequently reaches the pre-cut forest level. Vegetation succession begins with the removal of overstory vegetation, which initiates the growth of stress-tolerant vegetation species, and vegetation species composition tend to become associated with the forest at later stages of stand development [28,32,33,34]. 

In our study, the regression models were developed based on the moss and vascular plant cover at different *P. sylvestris* stands aged up to 130 years, including clear-cutting (see Figure 2). The obtained trends were comparable with those developed for the *Pinus contorta* Douglas. ex Loudon stands in Ref. [19]. The obtained trends indicated compositional stability of the species in the middle-aged stand, mainly between 40 and 60 years of age. Similarly, along with the decreased cover of vascular species, increased cover of moss species was reported. According to Ref. [11], the moss cover in the *P. sylvestris* stands during the stand rotation is not uniform. Although a decrease in moss cover in the first years after felling could be distinguished, the moss cover already recovers in young stands. Furthermore, the moss cover in the middle-aged stands tends to be higher than in the mature stands.

The current study found that the mosses were the dominant vegetation, covering more than 89% with the highest cover of *Hylocium splendens, Pleurozium schreberi* and *Ptilium crista-castrensis* in the *P. sylvestris* stands older than 30 years old (see Table 1 and Table 2). However, in the clear-cuts and young forest stands, representing an early successional stage of forest development, the mosses covered approximately 18–56%. The clear-cuts were characterized by the occurrence of new species, such as *Pohlia nutans*, *Polytrichum commune*, and *Polytrichum juniperinum*. Overall, a period of 10–15 years after clear-cutting can be considered as the period of successful recovery of the moss cover in the *Pinetum vaccinio-myrtilliosa* forest type. In accordance with the present results, previous studies have demonstrated that the cover of vascular plants was 14–40%, and the moss cover was 80–85% in the mature *P. sylvestris* stands growing at the *Vaccinio-myrtillosum* forest type [27,29]. At the early stage of stand development after clear-cutting, the increase or even doubling in vascular plant diversity is detected [34,35]. At later stages, the diversity of mosses and lichen species increases, but this process is longer and requires 5–6 years to find the significant increase [36]. Furthermore, interrelationships between different vegetation groups, such as mosses and vascular plants, are often studied as an indicator, having a great influence on the dominant plants in the ecosystem [37,38]. The mentioned study revealed that all studied mosses suppressed the regeneration of vascular plants, but differences between the moss species were identified [38]. Therefore, the reduction in vascular species richness often leads to a higher richness and diversity of non-vascular species [39]. 

Among all abiotic factors, intensive forest management, including clear-cutting, directly impacts light and soil nutrient availability, and these are the factors of great significance for recovering plant communities. The results of our study indicated that light was an important factor for herbs and dwarf shrubs. Therefore, the highest Ellenberg indicator values for light were obtained at the early stages of stand development, and these values decreased with the increase in stand age (see Table 4). However, the highest Ellenberg indicator values for light for the mosses were found in the relatively young to middle-aged stands compared to the clear-cuts and mature forests. Our data revealed that herbs and dwarf shrubs were more related to changes in soil temperature than to changes in moisture compared to mosses. These findings may be explained by the fact that the mosses are characterized as tolerant to shade stress and less dependent on soil moisture and fertility than vascular plants [40,41]. Overall, understory diversity increases with the increase in resource availability [42,43,44]. For example, the higher abundance of *Deschampsia flexuosa* on clear-cuts and the sites at early stages of forest development after clear-cutting was detected due to the increased nitrogen availability [45,46,47,48,49]. 

It is interesting to note that higher forest floor mass responded to the lower number of the moss species and tended to decrease the number of herbs and dwarf shrubs species (see Figure 3). However, the higher forest floor mass caused the lower percentage cover of herbs and dwarf shrubs but did not change the moss cover. It was already known from previous studies that moss colonization is associated with structural elements of the forest floor [50]. Overall, as noted by Ref. [13], most species typical for older forests could successfully survive in the clear-cuts. By increasing spatial diversity, clear-cutting creates the opportunity for populations of vegetation species other than bryophytes, which are more typical for the old-growth forest floor [10]. Our study also indicated large variability among the groups of the dominant herbs and dwarf shrubs, also moss species at different *P. sylvestris* sites (see Figure 4). For example, clearcutting greatly reduced *Vaccinium myrtillus* cover, which recovered slightly after 10 years and continued to increase in older stands. These results seem to be consistent with other studies which found a reduction in *Vaccinium myrtillus* abundance soon after clear-cutting and full recovery to mature stand level 60–80 years after clear-cutting [51]. Despite the ability to survive after forest harvesting, *Vaccinium myrtillus* was negatively affected by clear-cutting and this was well documented [47,49,52]. We also found the decrease in the cover of *Pleurozium schreberi* and *Hylocomium splendens* as a response to clear-cuttings, and these moss species are often defined as sensitive to final forest harvest [53].

When comparing the relations between the abundance of selected ground vegetation species and the chemical parameters of forest floor and upper mineral soil, the dominant species such as *Vaccinium myrtillus, Vaccinium vitis-idaea,* and *Hylocium splendens* showed moderately strong positive correlations with the soil pH values and total N concentrations (see Table 5). However, the soil chemical parameters such as total P, K, Ca, and Mg often responded in negative correlation to the mentioned species. No significant relations with soil nutrients were found for the *Pleurosium shreberi*, and low correlation was found for *Dicranum polysetum* and *Melampyrum pratense*. Accordingly, we identified species relation to chemical parameters as species-specific response. 

To conclude, the potential changes and variations in ground vegetation species indices in the *P. sylvestris* sites of different successional stages following clear-cuttings were investigated in this study. We have focused on the main maturity groups of *P. sylvestris* forest stands, typical for the hemiboreal forests: the clear-cuts of the first two years after felling, young stands of the 6, 10, and 15 years, middle-aged stands of 30 years, premature stands of 70 years, and mature stands 130 years old. This study has raised an important question about the relations between the ground vegetation species and chemical parameters of forest floor and upper mineral soil. The study findings indicated that the ground vegetation of the clear-cuts is more light-demanding, while the most shaded vegetation was obtained in the middle-aged stands. The ground vegetation species, obtained in the *P. sylvestris* stands assigned to the *Pinetum vaccinio-myrtillosum* forest type, showed the dependence on the parameters of the forest floor and upper mineral soil. These relations with the specific soil factors, along with the light factor, provide additional information in the evaluation of the ground vegetation diversity during the stand rotation following clear-cutting. It should be recognized that our data and insights do not give a full-scale view of ground vegetation development over the *P. sylvestris* stand rotation, but the results highlight the need to explore the links among a variety of forest ecosystem components. Additionally, the obtained specific findings could help to harmonize the aspects of intensive and close-to-nature forest management, taking into account the diversity of ground vegetation, and could help in decision making. Previous studies have shown that, for example, the close-to-nature system is not fully compatible with sustainable forest management in terms of biodiversity [5].

The trends identified in this study summarize several insights: firstly, changes in ground vegetation species composition and percentage cover following clear-cutting were identified as a relatively short-term process; secondly, forest ecosystems affected by clear-cutting, as an external disturbance, are forced to start a succession process, but vegetation changes tended to be related to the forest floor and upper mineral soil properties, the composition of the dominant forest-related species, and dependencies among the species of different groups; thirdly, new ground vegetation species occurring in the open-areas result in increased species richness, which has a positive effect on the overall ecosystem biodiversity. Therefore, clear-cutting system supports different species and a number of herbs and mosses at different stages of stand development. Forest ecosystems may lose certain vegetation species because some of them are limited by the frequency of disturbance rather than by the severity of disturbance [5]. 

## 4. Materials and Methods

### 4.1. Study Sites

Almost 34% of Lithuania is covered by forests [3]. Formally, Lithuanian forests belonged to the transitional hemiboreal forest zone of Europe with a prevalence of mixed deciduous and coniferous stands [54]. This study analyzes Scots pine (*Pinus sylvestris* L.) stands, which are the predominant coniferous forests (34.6%) in Lithuania [3].

The study on the understory vegetation in pure *Pinus sylvestris* forests at different successional stages following clear-cuttings was performed at the Trakai Regional Division of State Forest Enterprise in south-eastern Lithuania (54°38’16.19” N; 24°56’3.59” E) in June–September 2020 and 2021. The 1-year-old and 2-year-old clear-cuts, and 6-, 10-, 15-, 30-, 70-, and 130-year-old managed *P. sylvestris* stands were selected for this study. The 1-year-old clear-cut sites were established after the clear-cutting of the mature *P. sylvestris* forest, cultivated as even-aged stands, in 2019. The stems and logging residues, except stumps and roots, were removed from the cutting site. The 1-year-old clear-cut site was re-estimated the next year, obtaining the 2-year-old clear-cut site. More specifically, 2-year-old clear-cut site was reforested by 2-year-old *P. sylvestris* seedlings. Other study sites in the stands, representing different stand ages, were selected in the adjacent forest sites with a known forest management history. A pure *P. sylvestris* stand, which has recovered after clear-cutting managed mature stands, was chosen for the study. All selected sites were similar in climatic and edaphic conditions. The average annual temperature in Lithuania is 7.4 °C (1991–2020 climatic normal). The hottest month in Lithuania is July; the coldest is January. The average annual precipitation is 695 mm (1991–2020 climatic normal) [55]. All selected study sites were characterized by the *Pinetum vaccinio-myrtilliosa* forest type [28]; the oligotrophic mineral soil of a normal moisture regime (Nbl) [56]; and nutrient-poor sandy Arenosols with the light soil texture [57]. Typically, the underbrush species in the *Pinetum vaccinio-myrtilliosa* forest type are *Sorbus aucuparia* L., *Frangula alnus* Mill.*,* and *Juniperus communis* L. [28]. The mean characteristics are given in Table 7. 

### 4.2. Ground Vegetation Sampling and Analyses

The vegetation was assessed in July-August 2020 and 2021 when the vegetation cover was the highest and most diverse. For the assessment of ground vegetation, forest floor, and mineral soil parameters, a 2000 m^2^ circular plot was established at each site. In 2020, the permanent plot was established one year after clear-cutting (1-year-old clear-cut), and the investigations were repeated in the same plot 2 years after clear-cutting (2-year-old clear-cut). The temporary plots were established in the 6-, 10-, 15-, 30-, 70-, and 130-year-old *P. sylvestris* stands. The vegetation assessment was carried out on five sample plots of 10 × 10 m at each site, located in the directions of 0°, 90°, 180°, and 270° azimuths from the centre and one selected in the centre of the plot. At each site, the vegetation assessment was performed on 25 vegetation quadrates (in 1 × 1 m subplots). The frame of 1 dm^2^ square mesh was used to assess the cover (%) of each species in the vegetation quadrate visually. Then, the mean value of each species cover was calculated per site. The frequency of each species was determined as follows: the number of subplots in which the species was detected divided by a total number of subplots and multiplied by 100 [59]. The detailed scheme of the sample site, identifying the places for the ground vegetation assessment and soil sampling, was given in Ref. [60].

The ground vegetation species were recorded separately for moss layer and herb together with dwarf shrub layers < 0.5 m and > 0.5 m [61]. The tree layer was recorded for characteristics of the selected forest sites. The total richness of ground vegetation species was estimated as the total number of species obtained at each site, i.e., in the 1-year-old and 2-year-old clear-cuts, and 6-, 10-, 15-, 30-, 70-, and 130-year-old *P. sylvestris* stands. The mean richness of ground vegetation species was estimated as the mean of the species number obtained in every five sample plots within each study site (*n* = 5).

Aboveground biomass of ground vegetation was harvested within 25 × 25 cm squares from four subplots, located systematically in each of five sample plots within each site (1-year old and 2-year-old clear-cuts, and 6-, 10-, 15-, 30-, 70-, and 130-year-old *P. sylvestris* stands). All the living vegetation within a square was cut using scissors close to the ground surface. The harvested vegetation was put into plastic bags and transported to the laboratory. In the laboratory, litter and logging residues were removed from the samples, leaving only the living parts of the ground vegetation species. The moss species were separated into five groups: *Pleurozium schreberi*, *Hylocium splendens*, *Ptilium crista-castrensis*, *Dicranum* sp., and other mosses. The herbs and shrubs were separated into four groups: *Vaccinium myrtillus*, *Vaccinium vitis-idaea*, *Calluna vulgaris,* and other plants. The samples were oven-dried at 105°C to a constant mass and weighed.

### 4.3. Soil Sampling and Analyses

The organic layer (forest floor) was sampled within a 25 × 25 cm square using a metallic frame. The composite samples of the forest floor were obtained from four subsamples (*n* = 4), oven-dried at 105 °C to a constant mass, and weighted. Mineral soil was sampled from 0–10 cm by a metallic soil auger in August–September 2020. Four composite samples were combined from 10 subsamples collected systematically in each sample plot. The forest floor and mineral soil samples were analysed for pH, which was determined in a 0.01 M CaCl_2_ suspension (ISO 10390:2005); organic carbon (Corg) was determined using a dry combustion method with a total carbon analyzer Analytic Jena multi EA 4000, Jena, Germany, and total nitrogen (N) by the Kjeldahl method (ISO 11261). The concentration of mineral N was determined by the spectrometric method (ISO 14256-2) in 1 M KCl extraction. Soil available K_2_O and P_2_O_5_ were determined in ammonium lactate-acetic acid extractant [62] by the Shimadzu UV 1800 spectrophotometer (Shimadzu, Kyoto, Japan) and JENWAY PFP7 flame photometer, respectively. Soil available Ca and Mg were determined by atomic absorption spectrometer AAnalyst 200 (PerkinElmer, Waltham, MA, USA) (laboratory method LVP D-13:2016).

### 4.4. Calculations and Statistical Analysis

To assess the species diversity at different sites, the Shannon diversity index (H′) and equitability (E) were calculated [63]. The Shannon diversity index (*H′*) was calculated by the Equation (1):

(1) $$H'=-\sum \frac{n_{i}}{N}*ln\frac{n_{i}}{N}$$

here, *n_i_* is the coverage of the *i*th species; *N* is the total coverage of vegetation in the site.

Equitability (*E*) was calculated by the Equation (2):

(2) $$E=\frac{H’}{H{’}_{max}}$$

here H’max = ln(N); N is the total coverage of vegetation in the site.

Ellenberg’s original values, quantified originally for Central Europe, were used to identify the differences in the ecological performance of species under specific conditions in the clear-cuts and in the stands of different ages [45]. The average cover of vascular plants and mosses was used to calculate the mean weighted ecological scale values of Ellenberg by the Equation (3):

(3) $$E_{x}=\sum \frac{x_{i}\times n_{i}}{N}$$

here *Ex*—weighted average of environmental factor, *x_i_*—indicator value of *i*th species ecological factor, *n_i_*—the average coverage of *i*th species, and *N*—total coverage of species that are not indifferent to that ecological factor.

To calculate and draw a Venn diagram, we used an online program (http://bioinformatics.psb.ugent.be/webtools/Venn/, accessed on 30 August 2022). For the normality of the variables, the Shapiro–Wilk test was used, and the normality hypothesis was rejected with a 0.05 significance level. Therefore, the Kruskal–Wallis analysis of variance (ANOVA) test was used to ascertain the significant differences in variables between the sites. Pearson correlation was used to measure the strength of a linear correlation between two variables. Data are presented as means ± standard error (SE). Different letters next to the mean values are statistically significantly different at *p* < 0.05 between the sites. Statistical analyses were conducted using STATISTICA 12.0 (StatSoft. Inc., TULSA, OK, USA, 2007) software.

## Figures and Tables

**Table 1:** Mean cover (MC, %) and frequency (F, %), and prominence value (PV) of ground vegetation species at different *Pinus sylvestris* sites, including 1–2-year-old clear-cuts and 6–130 years old stands.

	**Clear-Cuts (Years)**						**Age of *Pinus sylvestris* Stand (Years)**																	
	**1**			**2**			**6**			**10**			**15**			**30**			**70**			**130**		
Taxa names	MC (%)	F (%)	PV	MC (%)	F (%)	PV	MC (%)	F (%)	PV	MC (%)	F (%)	PV	MC (%)	F (%)	PV	MC (%)	F (%)	PV	MC (%)	F (%)	PV	MC (%)	F (%)	PV
	**Herbs and dwarf shrubs lower than 50 cm**																							
*Agrostis tenuis* Sibith. (syn. *A. vulgaris*)													0.03 ± 0.02	15.00	0.12	0.34 ± 0.20	26.32	1.75						
*Anthoxanthum odoratum* L.				0.07 ± 0.04	12.82	0.24																		
*Arnica montana* L.										*														
*Betula pendula* Roth.	0.06 ± 0.05	7.50	0.17	0.14 ± 0.05	30.77	0.77	0.18 ± 0.11	20.00	0.80	1.65 ± 0.55	55.00	12.20	0.13 ± 0.07	15.00	0.48	0.04 ± 0.03	10.53	0.14						
*Calamagrostis arundinacea* (L.) Roth	0.04 ± 0.03	7.50	0.10				0.17 ± 0.15	15.00	0.67													0.01 ± 0.01	5.00	0.01
*Calluna vulgaris* (L.) Hull				0.07 ± 0.02	35.90	0.43	25.94 ± 3.40	100.0	259.4	23.38 ± 5.69	85.00	215.5	2.27 ± 1.25	25.00	11.33	0.05 ± 0.03	15.79	0.20						
*Carex* sp.				0.02 ± 0.02	2.56	0.04				0.03 ± 0.03	5.00	0.06	*			0.01 ± 0.01	5.26	0.01	0.02 ± 0.01	10.00	0.05			
*Chamaenerion angustifolium (L.) Holub* (syn. *Epilobium angustifolium* L.)				0.02 ± 0.01	5.13	0.04	0.01 ± 0.01	5.00	0.03															
*Chimaphila umbellata* (L.) W. P. C. Barton	0.04 ± 0.03	7.50	0.10																0.10 ± 0.10	5.00	0.22	0.04 ± 0.03	10.00	0.12
*Conyza canadensis* (L.) Cronquist				0.01 ± 0.01	2.56	0.01																		
*Convallaria majalis* L.	0.01 ± 0.01	2.50	0.02	0.14 ± 0.14	2.56	0.23							*											
*Convolvulus arvensis* L.				0.02 ± 0.02	2.56	0.03																		
*Epilobium parviflorum* Schreb.				0.03 ± 0.02	5.13	0.06				0.02 ± 0.02	5.00	0.04												
*Festuca ovino* L.				0.31 ± 0.23	5.13	0.71	2.10 ± 1.12	20.00	9.38	0.38 ± 0.31	10.00	1.19	1.11 ± 0.42	95.00	10.82	0.03 ± 0.03	5.26	0.06	0.056 ± 0.04	10.00	0.17			
*Festucapsammophila* (Hack. ex Čelak.) Fritsch	0.50 ± 0.29	12.50	1.76	0.26 ± 0.15	7.69	0.71				1.38 ± 0.66	30.00	7.53				4.73 ± 2.25	52.63	34.29	0.09 ± 0.06	10.00	0.27			
*Fragaria vesca* L.				0.06 ± 0.04	7.69	0.17				0.10 ± 0.07	10.00	0.32	0.03 ± 0.03	5.00	0.07	0.02 ± 0.02	5.26	0.04						
*Frangula alnus* Mill.	0.03 ± 0.02	7.50	0.08	0.05 ± 0.02	20.51	0.24				0.16 ± 0.08	10.00	0.51	*			0.07 ± 0.03	21.05	0.31						
*Galeopsis tetrahit* L.	0.03 ± 0.03	2.50	0.04	0.26 ± 0.14	10.26	0.84	0.01 ± 0.01	5.00	0.01							0.04 ± 0.03	10.53	0.12						
*Goodyera repens* (L.) R. Br.																0.05 ± 0.05	5.26	0.11	0.07 ± 0.03	25.00	0.33	0.10 ± 0.05	20.00	0.45
*Hieracium murorum* L.													0.12 ± 0.09	10.00	0.36									
*Hieracium pilosella* L.	0.04 ± 0.04	2.50	0.06																					
*Hypericum perforatum* L.				0.03 ± 0.03	7.69	0.08				0.01 ± 0.01	5.00	0.02												
*Hypochaeris radicata* L.				0.04 ± 0.03	5.13	0.10																		
*Juniperus communis* L.	*			*			*			0.30 ± 0.30	5.00	0.67	0.01 ± 0.01	5.00	0.02	*			0.01 ± 0.01			*		
*Knautia arvensis* (L.) Coult				0.10 ± 0.10	2.56	0.16	0.03 ± 0.03	5.00	0.06															
*Leontodon hispidus* L.				0.06 ± 0.06	2.56	0.10																		
*Lycopodium clavatum* L.	0.43 ± 0.23	15.00	1.66	0.14 ± 0.10	7.69	0.38													*			*		
*Luzula pilosa* (L.) Willd	0.35 ± 0.15	27.50	1.84	1.07 ± 0.21	76.92	9.38	0.02 ± 0.01	10.00	0.05	2.10 ± 1.20	45.00	14.07	0.22 ± 0.10	25.00	1.08	0.38 ± 0.09	63.16	2.99	0.18 ± 0.09	25.00	0.90	0.10 ± 0.10	5.00	0.22
*Maianthemum bifolium* (L.) F.W.Schmidt	*																							
*Melampyrum pratense* L.	0.01 ± 0.01	2.50	0.02	0.10 ± 0.04	15.38	0.38				1.53 ± 0.47	55.00	11.31	0.43 ± 0.16	70.00	3.56	0.16 ± 0.16	5.26	0.36	0.56 ± 0.20	40.00	3.54	*		
*Picea abies* (L.) H. Karst													0.04 ± 0.04	5.00	0.08	0.01 ± 0.01	5.26	0.02						
*Pinus sylvestris* L.	0.11 ± 0.04	27.50	0.59	0.64 ± 0.11	76.92	5.60	0.44 ± 0.24	30.00	2.41							0.02 ± 0.01	15.79	0.06						
*Plantago lanceolata* L.				0.01 ± 0.01	2.56	0.01																		
*Polygonum maculosa* L.				0.25 ± 0.25	2.56	0.40																		
*Populus tremula* L.	0.04 ± 0.04	2.50	0.06							0.07 ± 0.07	5.00	0.16	0.44 ± 0.17	35.00	2.60	0.02 ± 0.02	5.26	0.04						
*Potentilla arenaria* Borkh.	*			*																				
*Pteridium aquilinum* (L.) Kuhn	2.26 ± 0.95	15.00	8.73	2.37 ± 1.28	20.00	10.60	2.4 ± 1.70	30.00	13.15										0.15 ± 0.15	5.00	0.34			
*Rubus caesius* L.				0.41 ± 0.26	17.95	1.75				0.49 ± 0.30	20.00	2.19												
*Rubus idaeus* L.	0.13 ± 0.06	15.00	0.51	0.80 ± 0.30	30.77	4.43	1.79 ± 0.87	55.00	13.26	5.65 ± 1.65	65.00	45.53	0.18 ± 0.09	20.00	0.78	0.10 ± 0.07	10.53	0.33						
*Rumex acetosella* L.				0.16 ± 0.13	5.13	0.37	0.09 ± 0.06	15.00	0.34	0.08 ± 0.08	5.00	0.18	*											
*Salix caprea* L.	0.21 ± 0.12	7.50	0.57	0.31 ± 0.08	43.59	2.05																		
*Senecio vulgaris* L.	0.19 ± 0.18	5.00	0.42	4.83 ± 0.85	76.92	42.36	0.19 ± 0.11	25.00	0.93															
*Sorbus aucuparia* L.	0.07 ± 0.07	2.50	0.11										0.14 ± 0.14	5.00	0.30									
*Succisa pratensis* Moench				0.01 ± 0.01	2.56	0.01																		
*Trientalis europaea* L.	0.68 ± 0.22	45.00	4.53	1.12 ± 0.32	46.15	7.61				0.11 ± 0.05	25.00	0.55	0.04 ± 0.04	5.00	0.08									
*Trifolium repens* L.				0.01 ± 0.00	10.26	0.03																		
*Vaccinium myrtillus* L.	6.76 ± 0.83	95.00	65.90	6.64 ± 0.98	97.44	65.51	0.50 ± 0.28	30.00	2.72	7.83 ± 2.11	75.00	67.83	4.56 ± 1.23	65.00	36.74	13.0 ± 4.42	68.42	107.5	22.47 ± 2.25	100.0	224.7	10.58 ± 1.92	100.0	105.8
*Vaccinium vitis-idaea* L.	2.20 ± 0.34	80.00	19.63	1.89 ± 0.45	71.79	16.01	5.08 ± 2.14	65.00	40.96	13.42 ± 3.20	80.00	120.0	24.82 ± 2.44	100.0	248.2	6.30 ± 1.66	100.0	63.02	15.87 ± 1.96	100.0	158.7	5.81 ± 2.03	65.00	46.80
*Veronica officinalis* L.				0.31 ± 0.15	15.38	1.21	0.11 ± 0.07	20.00	0.50							0.02 ± 0.02	5.26	0.04						
*Verbascum thapsus* L.				*						0.02 ± 0.02	5.00	0.04												
*Viola canina* L.				0.02 ± 0.02	5.13	0.05				0.01 ± 0.01	5.00	0.03												
	**Herbs and dwarf shrubs higher than 50 cm**																							
*Betula pendula* Roth.							0.04 ± 0.04	6.00	0.10															
*Calamagrostis arundinacea* (L.) Roth							0.01 ± 0.01	5.00	0.02	0.35 ± 0.19	25.00	1.73	0.06 ± 0.06	5.00	0.12									
*Pinus sylvestris* L.							0.76 ± 0.76	1.00	0.76															
*Pteridium aquilinum* (L.) Kuhn	0.62 ± 0.36	8.00	1.75	0.65 ± 0.41	9.00	1.95																		
	**Mosses and lichens**																							
*Cirriphyllum piliferum* (Hed.) Grout.													28.98 ± 6.44	75.00	250.9									
*Cladonia fimbriata* (L.) Fr.							0.09 ± 0.03	40.00	0.60	0.48 ± 0.45	5.00	1.06	0.40 ± 0.40	5.00	0.89									
*Dicranum polysetum* Sw.	0.22 ± 0.13	12.50	0.78	0.18 ± 0.11	20.51	0.80	2.31 ± 1.95	25.00	11.55	4.75 ± 2.18	35.00	28.10	10.49 ± 4.55	100.0	104.9	3.74 ± 2.04	63.16	29.73	1.19 ± 0.68	30.00	6.49	0.07 ± 0.05	10.00	0.23
*Hylocium splendens* (Hedw.) Schimp.	13.57 ± 2.91	40.00	85.82	0.48 ± 0.24	17.95	2.03	1.61 ± 0.92	15.00	6.23	1.40 ± 0.59	40.0	8.85	0.83 ± 0.35	30.0	4.52	30.32 ± 6.41	78.95	269.4	13.71 ± 4.21	70.00	114.7	53.73 ± 8.00	100.0	537.3
*Pleurozium schreberi* (Brid.) Mitt.	37.18 ± 5.16	80.00	332.5	13.34 ± 2.82	84.62	122.7	5.19 ± 1.56	85.00	47.82	33.58 ± 5.09	90.0	318.5	17.50 ± 7.45	35.0	103.5	50.68 ± 7.18	100.0	506.8	67.78 ± 6.30	100.0	677.8	20.94 ± 6.21	75.00	181.4
*Pohlia nutans* (Hedw.) Lindb.				0.45 ± 0.24	25.64	2.27	13.89 ± 4.11	80.00	124.3	0.75 ± 0.34	25.00	3.75												
*Polytrichum commune* L.				0.01 ± 0.01	2.56	0.02	1.68 ± 0.62	60.00	13.02	6.55 ± 1.80	65.00	52.81	0.50 ± 0.26	20.00	2.24	2.87 ± 2.87	5.26	6.59	2.80 ± 1.07	55.00	20.73			
*Polytrichum juniperinum* Hedw.				0.01 ± 0.02	5.13	0.02																		
*Ptilium crista-castrensis* (Hedw.) De Not	5.13 ± 3.02	7.50	14.05	3.36 ± 1.54	33.33	19.39				4.13 ± 1.45	55.00	30.59	1.15 ± 1.10	10.00	3.64	5.56 ± 2.99	36.84	33.72	10.20 ± 3.88	60.00	79.01	14.11 ± 6.09	40.00	89.21
*Rhacomitrium canescens* (Hedw.) Brid.							0.52 ± 0.52	5.00	1.16	1.35 ± 0.70	25.00	6.75							0.30 ± 0.30	5.00	0.67			

* The species, detected on the border of the vegetation quadrate or at a maximum 50 cm distance from the vegetation quadrate.

**Table 2:** Mean cover (MC, %) and mean richness (MR, number) of the plant species, and forest floor cover (%) at different *Pinus sylvestris* sites, including 1–2-year-old clear-cuts and 6–130 years old stands.

	**Clear-Cut (Years)**				***Pinus sylvestris* Stand (Years)**											
	**1**		**2**		**6**		**10**		**15**		**30**		**70**		**130**	
	MC (%)	MR	MC (%)	MR	MC (%)	MR	MC (%)	MR	MC (%)	MR	MC (%)	MR	MC (%)	MR	MC (%)	MR
Mosses	56.2 ± 4.2	2.5 ± 0.2	18.1 ± 3.3	4.3 ± 0.3	25.1 ± 5.4	5.0 ± 0.7	53.0 ± 4.4	6.5 ± 0.5	59.8 ± 6.3	5.3 ± 0.3	92.5 ± 1.3	4.3 ± 0.3	96.0 ± 2.4	4.8 ± 0.6	88.9 ± 3.5	3.5 ± 0.3
Herbs, dwarf shrubs	14.5 ± 2.1	9.3 ± 1.3	23.2 ± 2.3	17.4 ± 0.7	39.0 ± 1.8	10.0 ± 1.6	59.0 ± 4.4	12.8 ± 1.6	34.6 ± 1.4	9.8 ± 0.8	24.1 ± 7.1	10.3 ± 1.0	39.6 ± 3.1	6.5 ± 0.3	16.6 ± 3.1	3.5 ± 0.7
Forest floor cover	95.8 ± 1.7	-	39.6 ± 4.7	-	86.6 ± 2.8	-	80.1 ± 4.8	-	99.8 ± 0.1	-	100.0 ± 0.0	-	100.0 ± 0.0	-	100.0 ± 0.0	-

**Table 3:** Shannon species diversity index (*H′*), and equitability (*E*) of ground vegetation species at different *Pinus sylvestris* sites, including 1–2-year-old clear-cuts and 6–130 years old stands. Different letters a, b, and c indicate significant differences among sites (*p* < 0.05).

	Clear-Cut (Years)		Age of *Pinus sylvestris* Stand (Years)					
	1	2	6	10	15	30	70	130
**Shannon diversity index (*H′*)**	1.58 ± 0.10 ^b^	2.04 ± 0.10 ^c^	1.52 ± 0.13 ^b^	1.85 ± 0.06 ^bc^	1.89 ± 0.06 ^bc^	1.67 ± 0.14 ^bc^	0.95 ± 0.09^a^	1.34 ± 0.06 ^b^
**Equitability (*E*)**	0.48 ± 0.04 ^a^	0.59 ± 0.03 ^ab^	0.55 ± 0.04 ^ab^	0.68 ± 0.01^b^	0.44 ± 0.08 ^a^	0.51 ± 0.03 ^a^	0.44 ± 0.05 ^a^	0.58 ± 0.03 ^ab^

**Table 4:** Ellenberg indicator values for the ground vegetation species at different *Pinus sylvestris* sites, including 1–2-year-old clear-cuts and 6–130 years old stands.

Ellenberg Indicator Values	Clear-Cut (Years)		Age of *Pinus sylvestris* Stand (Years)					
	1	2	6	10	15	30	70	130
	**Herbs and dwarf shrubs**							
Light (L)	5.4	6.0	7.3	6.5	5.3	5.0	5.0	5.0
Temperature (T)	4.9	5.0	5.0	5.1	4.9	5.5	5.4	5.9
Moisture (F)	4.6	4.7	4.3	4.2	4.0	4.1	4.0	4.0
pH	2.4	2.8	1.4	1.8	2.0	2.1	2.0	2.0
Nitrogen (N)	2.7	3.2	1.4	2.0	1.4	2.4	2.2	2.3
	**Mosses**							
Light (L)	5.8	5.6	5.5	6.4	6.5	5.9	5.8	5.7
Temperature (T)	2.9	2.8	1.3	2.9	3.0	2.9	2.9	2.8
Moisture (F)	4.2	4.4	4.2	5.1	4.6	4.2	4.3	4.3
pH	2.8	2.3	2.5	3.1	3.5	3.2	2.6	4.0

**Table 5:** Relations between the cover of ground vegetation species and forest floor (FF) and mineral soil (MS) chemical parameters (Pearson’s *r* and *p*-value).

		pH_CaCl__2_		Concentrations in Forest Floor (FF) and Mineral Soil at a Depth of 0–10 cm (MS)																					
				Organic C (g kg^–1^)		Total N (g kg^–1^)		Total P (mg kg^–1^)		Total K (mg kg^–1^)		Total Ca (mg kg^–1^)		Total Mg (mg kg^–1^)		P_2_O_5_ (mg kg^–1^)		K_2_O (mg kg^–1^)		Mobile Ca (mg kg^–1^)		Mobile Mg (mg kg^–1^)		Mineral N (mg kg^–1^)	
		*r*	*p*	*r*	*p*	*r*	*p*	*r*	*p*	*r*	*p*	*r*	*p*	*r*	*p*	*r*	*p*	*r*	*p*	*r*	*p*	*r*	*p*	*r*	*p*
***Vaccinium*** ***myrtillus***	FF	0.687	0.000	−0.581	0.000	0.697	0.000	−0.704	0.000	−0.701	0.000	−0.700	0.000	−0.532	0.002	n.d.*	n.d.	n.d.	n.d.	n.d.	n.d.	n.d.	n.d.	n.d.	n.d.
	MS	0.389	0.033	n.s.**	n.s.	n.s.	n.s.	−0.698	0.000	−0.751	0.000	−0.675	0.000	−0.731	0.000	−0.580	0.000	−0.721	0.000	−0.724	0.000	n.s.	n.s.	0.469	0.009
***Vaccinium*** ***vitis-idaea***	FF	0.560	0.001	−0.497	0.004	0.447	0.010	−0.643	0.000	−0.644	0.000	−0.628	0.000	−0.513	0.003	n.d.	n.d.	n.d.	n.d.	n.d.	n.d.	n.d.	n.d.	n.d.	n.d.
	MS	0.685	0.000	0.607	0.000	0.666	0.000	−0.665	0.000	−0.688	0.000	−0.657	0.000	−0.697	0.000	−0.597	0.001	−0.693	0.000	−0.611	0.000	−0.486	0.006	0.453	0.012
***Luzula*** ***pilosa***	FF	0.504	0.004	−0.485	0.007	0.494	0.006	−0.577	0.000	−0.580	0.001	−0.551	0.002	−0.420	0.021	n.d.	n.d.	n.d.	n.d.	n.d.	n.d.	n.d.	n.d.	n.d.	n.d.
	MS	0.413	0.023	0.581	0.001	0.652	0.000	−0.568	0.001	−0.590	0.001	−0.529	0.003	−0.587	0.001	−0.507	0.004	-	−0.565	−0.553	0.002	n.s.	n.s.	0.416	0.023
***Melampyrum*** ***pratense***	FF	n.s.	n.s.	n.s.	n.s.	n.s.	n.s.	n.s.	n.s.	−0.442	0.015	−0.420	0.021	n.s.	n.s.	n.d.	n.d.	n.d.	n.d.	n.d.	n.d.	n.d.	n.d.	n.d.	n.d.
	MS	0.468	0.009	n.s.	n.s.	0.510	0.004	−0.443	0.014	0.457	0.011	−0.403	0.028	−0.453	0.012	−0.393	0.032	−0.433	0.017	−0.444	0.001	n.s.	n.s.	n.s.	n.s.
***Rubus*** ***idaeus***	FF	0.564	0.001	−0.390	0.035	n.s.	n.s.	−0.439	0.015	−0.446	0.014	−0.414	0.023	n.s.	n.s.	n.d.	n.d.	n.d.	n.d.	n.d.	n.d.	n.d.	n.d.	n.d.	n.d.
	MS	n.s.	n.s.	n.s.	n.s.	0.510	0.004	−0.443	0.014	−0.453	0.012	−0.409	0.025	−0.409	0.252	−0.401	0.029	−0.430	0.018	−0.425	0.019	n.s.	n.s.	0.421	0.020
***Pleurozium*** ***schreberi***	FF	0.384	0.036	n.s.	n.s.	n.s.	n.s.	n.s.	n.s.	n.s.	n.s.	n.s.	n.s.	n.s.	n.s.	n.d.	n.d.	n.d.	n.d.	n.d.	n.d.	n.d.	n.d.	n.d.	n.d.
	MS	n.s.	n.s.	n.s.	n.s.	n.s.	n.s.	n.s.	n.s.	n.s.	n.s.	n.s.	n.s.	n.s.	n.s.	n.s.	n.s.	n.s.	n.s.	n.s.	n.s.	−0.365	0.047	n.s.	n.s.
***Dicranum*** ***polysetum***	FF	0.391	0.033	n.s.	n.s.	0.471	0.007	n.s.	n.s.	−0.406	0.026	0.386	0.035	n.s.	n.s.	n.d.	n.d.	n.d.	n.d.	n.d.	n.d.	n.d.	n.d.	n.d.	n.d.
	MS	n.s.	n.s.	n.s.	n.s.	0.406	0.026	−0.410	0.029	−0.424	0.020	−0.391	0.033	−0.423	0.020	n.s.	n.s.	−0.413	0.023	−0.401	0.029	−0.417	0.022	n.s.	n.s.
***Hylocium*** ***splendens***	FF	0.509	0.004	0.568	0.001	n.s.	n.s.	−0.556	0.001	−0.572	0.001	−0.509	0.004	0.465	0.010	n.d.	n.d.	n.d.	n.d.	n.d.	n.d.	n.d.	n.d.	n.d.	n.d.
	MS	0.395	0.031	n.s.	n.s.	0.508	0.004	−0.563	0.001	−0.577	0.001	−0.436	0.016	−0.555	0.002	−0.480	0.007	−0.578	0.001	−0.579	0.001	−0.531	0.003	0.441	0.015
***Ptilium crista-*** ***castrensis***	FF	n.s.	n.s.	0.436	0.016	0.465	0.010	−0.496	0.005	−0.500	0.005	−0.486	0.006	−0.386	0.035	n.d.	n.d.	n.d.	n.d.	n.d.	n.d.	n.d.	n.d.	n.d.	n.d.
	MS	n.s.	n.s.	0.502	0.005	0.590	0.001	−0.491	0.006	−0.523	0.003	−0.456	0.011	−0.512	0.004	−0.419	0.022	−0.500	0.005	−0.515	0.004	n.s.	n.s.	n.s.	n.s.

* no data in the forest floor; ** not significant.

**Table 6:** Aboveground biomass (kg ha^–1^; mean ± SE) of the mosses and herbs and dwarf shrubs at different *Pinus sylvestris* sites, including 1–2-year-old clear-cuts and 6–130 years old stands. Different letters a, b, and c indicate significant differences among sites (*p* < 0.05).

	Clear-Cut (Years)		Age of *Pinus sylvestris* Stand (Years)					
	1	2	6	10	15	30	70	130
Mosses (kg ha^–1^)	5269 ± 634 ^c^	1063 ± 302 ^a^	1153 ± 248 ^a^	3790 ± 54 ^b^	2374 ± 973 ^ab^	3136 ± 830 ^b^	1965 ± 931 ^ab^	5353 ± 964 ^c^
Herbs and dwarf shrubs (kg ha^–1^)	1207 ± 352 ^b^	7120 ± 502 ^c^	309 ± 214 ^a^	10,650 ± 353 ^d^	12993 ± 823 ^d^	1148 ± 420 ^b^	7094 ± 1248 ^c^	1848 ± 191 ^b^
Total (kg ha^–1^)	6476 ± 725 ^c^	8183 ± 586 ^d^	1462 ± 328 ^a^	14440 ± 357 ^e^	15368 ± 1275 ^e^	4284 ± 930 ^b^	9059 ± 1557 ^d^	7201 ± 983 ^cd^

**Table 7:** Characteristics of the *P. sylvestris* L. forests (Mean tree height (H), DBH, basal area, and characteristics of underbrush were measured during this survey according to Ref. [58]; stand volume data were obtained from the Lithuanian State Forest Cadastre database (2021).

Study Site (Years)	Tree Species Composition* (%)	Mean Tree H (m)	Mean Tree DBH (cm)	Stand Volume (m^3^ ha^– 1^)	Basal Area (m^2^ ha^– 1^)	Underbrush		
						Species Composition (%) *	Density (%)	Mean H (m)
**Clear-cuts**								
1	-	-	-	-	-	43Gw+23P+14Rw+12B+8A	50	0.1
2	90 P + 10 S	0.2	-	-	-	39Gw+38P+18B+5Bc	40	0.2
***Pinus sylvestris*** **stands**								
6	100 P	1.6	0.4	10	-	90 B + 10 P	46	1.0
10	100 P	4.5	5.0	58	16	55B+39Bc+3S+3A	30	1.3
15	100 P	7.0	9.0	56	14	56A+17Bc+11J+11P+5B	25	1.3
30	90 P + 10 S	15.0	15.0	165	28	55S+36J+9Bc	20	1.5
70	100 P	25.0	25.0	294	32	60Bc+40J	10	2.0
130	100 P	29.0	38.0	465	36	50S+30J+20Bc	20	2.1

* P—P. sylvestris; S—Picea abies (L.) H. Karst.; Gw—Salix caprea L.; Rw—Sorbus aucuparia L.; B—Betula pendula Roth; A—Populus tremula L.; Bc—Frangula alnus Mill.; J—Juniperus communis L.

## Data Availability

Not applicable.
